# Invasive *Candida bovina* Infection, France 

**DOI:** 10.3201/eid2603.191371

**Published:** 2020-03

**Authors:** Kévin Brunet, Alida Minoza, Blandine Rammaert, Vincent Portet-Sulla, Florent Hubert, Jean-Claude Lorenzo, Marie-Hélène Rodier, Estelle Cateau

**Affiliations:** Poitiers University Hospital, Poitiers, France (K. Brunet, A. Minoza, B. Rammaert, V. Portet-Sulla, F. Hubert, J.-C. Lorenzo, M.-H. Rodier, E. Cateau);; INSERM U1070, Poitiers (K. Brunet, B. Rammaert);; University of Poitiers, Poitiers (K. Brunet, B. Rammaert, M.-H. Rodier, E. Cateau);; CNRS 7267, Poitiers (M.-H. Rodier, E. Cateau)

**Keywords:** healthcare-associated infection, candidemia, candidiasis, emerging fungal disease, fungal infections, Candida bovina, zoonoses, matrix-assisted laser desorption/ionization time-of-flight mass spectrometry, MALDI-TOF, France, fungi

## Abstract

New *Candida* species such as *Candida auris* have emerged recently as important invasive fungal diseases. We report a case of *C. bovina* bloodstream infection in a 94-year-old patient in France. The species led to identification issues because it was misidentified by phenotypic and matrix-assisted laser desorption/ionization time-of-flight mass spectrometry methods.

A 94-year-old patient in France with no remarkable medical history was hospitalized because of a fall. During hospitalization, the patient had digestive disorders with postprandial regurgitations, leading to undernutrition. At hospital day 24, we performed an abdominal computed tomography scan, which indicated a dolicho-mega esophagus. Because of undernutrition, we introduced parenteral nutrition through peripheral venous catheter on day 36. We performed endoscopic pneumatic dilation to treat achalasia of the esophagus lower sphincter on day 51. One day later (day 52), the patient had a fever, triggering a bloodstream culture, which was positive after 24 hours (day 53) for *Streptococcus mitis/oralis* and *S. gordonii*. Parenteral nutrition was stopped, the peripheral catheter was removed, and the patient received piperacillin/tazobactam. On day 56, a catheter culture was positive for *S. mitis/oralis* and for a yeast. We identified this yeast by using matrix-assisted laser desorption/ionization time-of-flight (MALDI-TOF) mass spectrometry with the Vitek MS 3.2 system (bioMérieux, https://www.biomerieux-diagnostics.com), which revealed *Candida slooffiae* with a maximal score of probability. 

We performed bloodstream cultures on day 57. Aerobic culture was positive on day 58 for a yeast again, which we identified as *C. slooffiae* by using Vitek MS, and anaerobic culture was positive for *S. anginosus*. We initiated treatment with caspofungin (70 mg loading dose, then 50 mg/d). Three days later, the patient was nonpyretic, and the inflammatory syndrome regressed. Results of blood cultures performed on day 63 were negative. Caspofungin was continued for 14 days after the negative culture, and no valvular disease or cardiac failure was noted. Moreover, funduscopic examination revealed no abnormalities. 

Although Vitek MS indicated a MALDI-TOF mass spectrometry high score of identification, we performed sequencing of *Candida* 18S ribosomal DNA to confirm identification of this rare species. This sequencing identified the isolate as *C. bovina* (GenBank accession no. MN704805). Sequence analysis showed 100% similarity with ribosomal DNA sequences (internal transcribed spacer [ITS] 1–5.8S through ITS2–28S) of 2 *C. bovina* isolates from 2 different collection centers (GenBank nos. KY103626.1 and KP132306.1). After 90 days of monitoring, the patient showed improvement and had no relapse of infection.

The prevalence of healthcare-associated *Candida* bloodstream infections has increased over recent decades in many countries, mainly because of use of immunosuppressive drugs and critical care therapies (*1*). Among involved species, non–*C.* *albicans Candida* have emerged, especially because of use of fluconazole as a prophylactic drug, which promotes emergence of fluconazole nonsusceptible *Candida* (*1**,**2*). Moreover, new *Candida* species have emerged, such as *C. auris* (*3*). Enhanced use of antifungal agents in susceptible patients might lead to an increase of candidiasis because of uncommon *Candida* species. The ability to identify and treat infections with these organisms, which might have high antifungal MICs, is critical.

*C. bovina*, first described in 1957 from bovine cecum (*4*), is a member of the *Kazachstania* (*Arxiozyma*) *telluris* complex, which includes *C.* (*Kazachstania*) *bovina*, *C.* (*Kazachstania*) *pintolopesii*, *C.* (*Kazachstania*) *sloofiae*, *K. heterogenica*, and *K.* *telluris* (*5*). Members of the *K. telluris* complex are known to cause infections in rodents and birds and, less frequently, in horses, pigs, and cows (*5*). *C. bovina* has been isolated from cows, birds, and, rarely, humans (*5*), but human disease has not been previously reported.

*C. bovina* yeast grows on usual yeast culture media (i.e., Sabouraud, Sabouraud gentamicin chloramphenicol, and potato dextrose agar) in 24 hours and forms small colonies. This yeast was inhibited by cycloheximide and was found to be white on a chromogenic CAN2 medium (bioMérieux). Attention must be paid to misidentification through use of MALDI-TOF mass spectrometry technology, which appears unable to distinguish yeasts inside the *Kazachstania (Arxiozyma) telluris* complex. Vitek MS 3.2 identified the organism as *C. slooffiae*; identification was *K. telluris* by using Bruker Biotyper MS 4.1.80 (Bruker Daltonics, https://www.bruker.com). Phenotypic identification using the Vitek2 YST card (bioMérieux) was unable to identify fungi. Sequencing of ITS1 and ITS4 (18S ribosomal DNA) must be performed and appears able to effectively differentiate species inside this complex.

We evaluated MICs by using European Committee on Antimicrobial Susceptibility Testing broth microdilution (*6*) (Table). Among azoles, fluconazole showed MICs of 2 mg/L, considered susceptible because the breakpoint for all non–*C. glabrata Candida* is 2 mg/L. However, this MIC is higher than those of common *Candida*, such as *C. albicans* (MIC_90_ <0.25 mg/L) (*7*). For other antifungal agents, interpretation of MICs is complicated in the absence of specific clinical breakpoints. However, MICs were comparable to those of common species (*7*). In the case of this patient, catheter removal and caspofungin treatment led to recovery.

**Table:** Antifungal susceptibility of *Candida bovina* from a patient in France, evaluated by using EUCAST broth microdilution*

Antifungal agent	MIC, mg/L
Fluconazole	2
Posaconazole	≤0.016
Voriconazole	0.03
Isavuconazole	0.015
Amphotericin B	0.03
Caspofungin	0.06
Micafungin	0.03

*EUCAST, European Committee on Antimicrobial Susceptibility Testing.

In conclusion, this case illustrates the emergence of an uncommon *Candida* species about which laboratory staff are advised to be aware. These new species might lead to issues with identification and antifungal susceptibility. Clinicians should remain skeptical of infections with uncommon yeast species and consider confirmation through DNA sequencing.
